# Nitric Oxide and Mechano-Electrical Transduction in Cardiomyocytes

**DOI:** 10.3389/fphys.2020.606740

**Published:** 2020-12-15

**Authors:** Hannah E. Boycott, My-Nhan Nguyen, Besarte Vrellaku, Katja Gehmlich, Paul Robinson

**Affiliations:** ^1^Division of Cardiovascular Medicine, Radcliffe Department of Medicine, University of Oxford, John Radcliffe Hospital, Oxford, United Kingdom; ^2^Division of Cardiovascular Medicine, Radcliffe Department of Medicine and British Heart Foundation Centre of Research Excellence Oxford, University of Oxford, Oxford, United Kingdom; ^3^Institute of Cardiovascular Sciences, University of Birmingham, Birmingham, United Kingdom

**Keywords:** nitric oxide, nitric oxide synthase, cytoskeleton, cellular stress, mechano-electrical transduction, stretch, cardiomyocyte, calcium

## Abstract

The ability^§^ of the heart to adapt to changes in the mechanical environment is critical for normal cardiac physiology. The role of nitric oxide is increasingly recognized as a mediator of mechanical signaling. Produced in the heart by nitric oxide synthases, nitric oxide affects almost all mechano-transduction pathways within the cardiomyocyte, with roles mediating mechano-sensing, mechano-electric feedback (via modulation of ion channel activity), and calcium handling. As more precise experimental techniques for applying mechanical stresses to cells are developed, the role of these forces in cardiomyocyte function can be further understood. Furthermore, specific inhibitors of different nitric oxide synthase isoforms are now available to elucidate the role of these enzymes in mediating mechano-electrical signaling. Understanding of the links between nitric oxide production and mechano-electrical signaling is incomplete, particularly whether mechanically sensitive ion channels are regulated by nitric oxide, and how this affects the cardiac action potential. This is of particular relevance to conditions such as atrial fibrillation and heart failure, in which nitric oxide production is reduced. Dysfunction of the nitric oxide/mechano-electrical signaling pathways are likely to be a feature of cardiac pathology (e.g., atrial fibrillation, cardiomyopathy, and heart failure) and a better understanding of the importance of nitric oxide signaling and its links to mechanical regulation of heart function may advance our understanding of these conditions.

## Introduction

The heart is a mechanically active organ. With every heartbeat cardiac cells are subjected to various mechanical forces, including stretch, shear, and strain (Waldman et al., 1985). Moreover, the mechanical forces experienced by the heart are not constant, varying with posture, exercise, stimulants (such as caffeine), and emotional state. The way that the heart responds to alterations in the mechanical environment has been the subject of investigation for decades. There is also huge medical relevance given that cardiac pathologies are often accompanied by a change in the mechanical environment where hypertrophy or increased fibrosis causes altered intrinsic tension in the heart chamber walls (Abrahams et al., 1987; Weber et al., 1988; Conrad et al., 1995; De Jong et al., 2011).

In this review we discuss the role of nitric oxide as a key modulator and of various mechanical forces in the cardiomyocyte. The production of NO is influenced by mechanical forces and NO is known to be a key mediator in mechano-chemo-transduction (MCT) during cardiac contraction. This provides therapeutic targets for treating mechanical stress-induced Ca^2+^ dysregulation, arrhythmias, and cardiomyopathy. Current understanding of the interaction between NO and MCT signaling pathways in the heart is incomplete, particularly whether mechanically ion-sensitive channels are regulated by NO, and how this affects the cardiac action potential.

Each defined mechanical force is governed by a defined set of protein interactors and intrinsic physiological processes:

1. Overall contractile force in cardiac muscle is generated principally by the actomyosin ATPase interactions of the sarcomeric thick and thin filaments, whilst the degree of acto-myosin interaction is controlled by Ca^2+^ binding to the regulatory troponin/tropomyosin complex on the actin filament.
2. The passive stiffness of the cardiomyocyte is generated by the giant protein titin along the intermediate filament of the sarcomere and extracellular collagen, where the amount and degree of cross linking within the collagen matrix can stiffen the myocardium in certain pro-fibrotic disease pathologies leading to diastolic dysfunction. Stiffness can also be influenced to a smaller extent by changes in the cytoskeletal tubulin network.
3. Myocardial stretch is a force on the cardiomyocyte in response to the hemodynamic load within the chambers of the heart. Molecular responses to increased stretch is termed stretch sensing and allows the heart to respond to stimulatory homeostasis of the sympathetic nervous system during periods of physiological arousal or compromise such as exercise or hemorrhage, to prevent circulatory congestion or failure.
4. Shear stress occurs as a result of fluid shear due to blood flow, and when laminar sheets of cardiomyocytes slide relative to each other when the heart contracts. This causes cardiomyocyte deformation and shear forces.
5. Preload/myocardial strain refers to the degree to which the myofilaments are initially stretched during ventricular filling. The sarcomere is able to quickly respond to alterations in preload due to the highly regular arrangement of its filaments to produce more contractile force and thereby increase the stroke volume of the heart in a process defined as the Frank-Starling mechanism (discussed in more detail below).
6. Afterload/myocardial stress is determined by the work the ventricular myocardium is required to perform in order to eject blood into circulation against the systemic vascular resistance. Changes in afterload affect the calcium handling and contractility, which may underlie the Anrep effect.

The intrinsic ability of the myocardium to adapt to increased stretch by increasing contractile force is embodied by the Frank-Starling mechanism (Frank, 1899; Starling, 1918; Sagawa et al., 1990), which links stroke volume and end diastolic pressure. The consequences of this are that increased diastolic filling (preload) leads to a stronger contraction and an increased stoke volume (Lakatta, 1987). The Frank Starling mechanism is underpinned by spatial rearrangement of sarcomeric filaments, bringing actin, and myosin filaments to closer proximity to increase the myosin duty ratio, when the sarcomere lengthens as the myocyte stretches, and increases calcium sensitivity of myofilaments. This leads to enhanced cross bridge formation, as well as stretch activated titin unfolding (Fukuda et al., 2009; Ait-Mou et al., 2016). The rapid response of the mechanism is accompanied by a slower, less pronounced increase in contractility known as the Anrep effect (von Anrep, 1912), where contractility is influenced by an increase in ventricular inotropy as a consequence of afterload-induced activity of tension dependent Na^+^/H^+^ exchangers (Cingolani et al., 2005). The Frank-Starling mechanism has been shown to be modulated by nitric oxide (NO) which affects the myofilament responsiveness to Ca^2+^ (Prendergast et al., 1997). In addition, the Anrep effect is modulated by NO; increased NO results in an increase in intracellular Ca^2+^ sparks (Petroff et al., 2001).

The role of the molecular gas NO in the cardiovascular system is well established, where it regulates a multitude of cellular processes. Endothelial cells synthesize and release NO which mediates diverse effects including vessel tone, hemostasis, blood pressure and vasculature remodeling (Naseem, 2005). The importance of NO for cardiomyocyte function is well recognized, with roles in ion channel regulation, Ca^2+^ homeostasis, contractility, energetics, cell growth, and survival (Shah and MacCarthy, 2000; Seddon et al., 2007).

Nitric oxide is synthesized by a family of enzymes known as nitric oxide synthases (NOS), which produce NO as they catalyze the conversion of L-arginine to L-citrulline. There are three isoforms of NOS (NOS1-3) each encoded by a specific gene. In the cardiovascular system “neuronal” NOS (nNOS or NOS1) and “endothelial” NOS (eNOS or NOS3) are expressed in cardiomyocytes (Feron et al., 1996); eNOS is expressed in the endothelium and fibroblasts. nNOS can also be found in the neurons which innervate the heart (Choate et al., 2008). The third isoform, “inducible” NOS (iNOS or NOS2) is produced in most cell types in response to stimuli such as hypoxia or cytokines [e.g., Tumor Necrosis Factor-α (TNFα)] (Shah, 2000).

Nitric oxide synthase-derived NO exerts its effects in a variety of ways. Firstly, NO is able to post-translationally modify target proteins primarily through the addition of a nitroso group, to the sulfhydryl side chain of cysteine, termed-S-nitrosylation (Kovacs and Lindermayr, 2013). Such modification results in an alteration in the target protein’s function (Gonzalez et al., 2009). The effects of direct NO modification of target proteins are limited by the relatively short diffusion distance of the molecule. Cellular carrier molecules such as S-nitrosoglutathione mediate longer range NO-signaling by acting as a carrier and a donor, transferring NO onto more distal targets (Such-Miquel et al., 2018). Exogenous application of glutathione exacerbates ventricular arrhythmias induced by stretch, which has been proposed to be a potential link between NO cycling and mechanical response (Such-Miquel et al., 2018). NO also activates the soluble guanylate cyclase (sGC)/cyclic guanosine monophosphate (cGMP)/protein kinase G (PKG)-dependent phosphorylation pathway. Activation of this pathway results in the phosphorylation of target proteins, to inhibit the mitogen-activated protein kinase kinase/extracellular signal-regulated kinase (MEK1/2/ERK1/2) pathway and activate c-Jun N-terminal kinase (JNK)1, 2, and 3 pathways, the ultimate result being cardio-protection and down regulation of genes involved in hypertrophy and regulation of genes involved in apoptosis (Pilz and Casteel, 2003). It has been speculated that the majority of nitrosylation is carried out by nNOS, whereas the cGMP pathway is primarily mediated by eNOS (Ziolo, 2008).

An imbalance in NO bioavailability has been implicated in the pathology of several cardiac pathologies and understanding of the processes underlying this dysregulation has been the subject of intense research. In pathological conditions NOS can become uncoupled leading to an increase in reactive oxygen species production rather than the physiological production of NO (reviewed in Roe and Ren, 2012). Uncoupling occurs when the tightly controlled flow of electrons through the NOS enzyme is redirected by the dissociation of the ferrous-dioxygen complex (Forstermann and Munzel, 2006), and has been linked to reduced levels of the cofactor tetrahydrobiopterin (BH4). Reduced levels of BH4 leading to NOS uncoupling are contributing factors in several pathologies, including atrial fibrillation, hypertension, ischemia/reperfusion injury and overload-induced heart failure (reviewed in Alkaitis and Crabtree, 2012). Increased superoxide production following NOS uncoupling increases the oxidative burden on the heart and affects cardiac performance (Singal et al., 1998).

Nitric oxide production is increased in response to various mechanical forces, a phenomenon which is of particular importance for cardiovascular function. For example, studies using NO sensitive dyes have shown that stretch of ventricular cardiomyocytes induces NO release (Petroff et al., 2001; Shim et al., 2017a). Furthermore, swelling of cardiomyocytes induced by hypotonic solution leads to an increase in NO production (Gonano et al., 2014). The response of cardiomyocytes to mechanical stimuli is of particular interest given the importance of the force-response phenomenon to heart function. For these reasons NO function in physiology and pathology has been the subject of intense investigation for several decades. Other redox molecules, particularly NADPH oxidase 2 and X-ROS signaling, are also altered in response to mechanical stimuli and have been reviewed elsewhere (Prosser et al., 2013; Ward et al., 2014). It has become clear from the literature over the last 10 years that NO appears to be the major modulator of mechano-transduction pathways in the heart via direct nitrosylation or activation of PKG. Here, we review these latest findings, with particular emphasis on three key areas: mechano-sensing, ion channel activity and calcium homeostasis.

## Mechano-Sensing by the Cytoskeleton

In order to be transduced into a biological response, mechanical signals must be effectively sensed by the cell and transduced into meaningful signals. This process is mediated either by ion channels which can alter their activity in response to mechanical signals, or by the cytoskeleton which senses changes in mechanical load and alters cell function accordingly. The cytoskeleton is a network of tubules and filaments that aids in maintaining the structural and mechanical integrity of the cardiomyocyte (Sequeira et al., 2014). Importantly, the cytoskeleton is also involved in mechano-transduction in response to mechanical stress through its interaction with several membrane-associated proteins, which includes proteins associated with the integrin complex, dystrophin-glycoprotein complex (DGC), LIM domain proteins, and titin. Nitric oxide signaling has the ability to modulate cytoskeletal structure and function. For instance, the myofilament response to Ca^2+^ is reduced by high concentrations of NO via cGMP-dependent activation of PKG (Vila-Petroff et al., 1999) which can in turn regulate the expression of genes such as atrial natriuretic peptide (ANP), brain natriuretic peptide (BNP), β-Myosin and cardiac actin (Pilz and Casteel, 2003). The importance of NO signaling for cytoskeletal remodeling stems from post-translational modifications of various cytoskeletal and membrane-associated proteins, which influence cardiomyocyte contractility and/or relaxation.

### Integrins and the Integrin Complex

The integrin complex is a group of cytoskeletal proteins essential for not only facilitating cellular adhesion, but also the sensing and integration of mechanical signals. Integrins are heterodimeric transmembrane receptors which associate the extracellular matrix with the actin cytoskeleton, whereby they transmit mechanical signals to the cytoskeleton (Wang et al., 1993). In cardiomyocytes, integrins contribute to preservation of cardiac function and have the ability to modulate the mechanical and electrical coupling in the heart (Valencik et al., 2006). The impact of NO signaling on cardiac integrins has been examined via the utilization of ligands containing the amino acid sequence of Arg-Gly-Asp (RGD) binding motif. Application of this peptide has been reported to modulate L-type Ca^2+^ currents (Wu et al., 1998) and affect Ca^2+^ release from the sarcoplasmic reticulum (Chan et al., 2001). In neonatal rat cardiomyocytes, pre-treatment with the NO donor SNAP enhanced Ca^2+^ release, whilst pre-treatment with the NOS inhibitor ng-nitroarginine methyl ester (L-NAME) abolished the release following integrin stimulation by RGD peptide (van der Wees et al., 2006), suggesting that stimulation of integrins promotes NO-mediated Ca^2+^ release from the sarcoplasmic reticulum. Further, this may be mediated by focal adhesion kinase (FAK) activation, as incubation of cardiomyocytes with the RGD peptide increased phosphorylation of FAKs, whilst incubation with SNAP induced phosphorylation of FAK (van der Wees et al., 2006). In addition to integrins modulating Ca^2+^ homeostasis via NO-signaling, it has been reported that the stimulatory effects of NO directly regulate integrin expression via cGMP signaling (Zhan et al., 2018).

**FIGURE 1:**
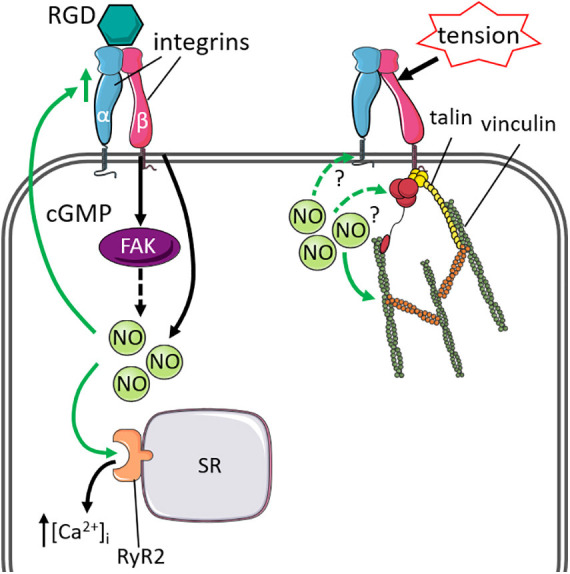
Cytoskeletal proteins linked to integrin that influence or are influenced by nitric oxide signaling and mechanical stimuli. **Left:** Stimulation of integrins by ligands containing the Arg-Gly-Asp motif (RGD) increased nitric oxide (NO)-mediated intracellular Ca^2+^ [(Ca^2+^)_*i*_] release from the sarcoplasmic reticulum (SR), via activation of ryanodine receptor 2 (RyR2). The stimulatory effects of NO can also regulate integrin expression via cGMP-signaling. Solid green lines indicate effect by NO. **Right:** Integrins form a complex with talin and vinculin to stabilize the actin-cytoskeleton under mechanical stress. The effect of cardiac NO signaling on the integrin-talin-vinculin complex remains to be investigated (green dashed lines).

Other components of the integrin complex are also important for regulating mechano-sensing and mechano-transduction. Talin and vinculin are intracellular cytoskeletal proteins that interact with both integrins and the actin cytoskeleton at focal adhesions. In response to external forces, talin, and vinculin alter their conformations to reinforce and stabilize the integrin-actin cytoskeleton connection (del Rio et al., 2009). The impact of NO signaling on talin and vinculin has been studied in skeletal muscle. Increased NO signaling in response to mechanical stimuli mediates skeletal talin and vinculin expression via a PKG-dependent manner, and prevents calpain-proteolytic degradation of talin (Koh and Tidball, 2000; Zhang et al., 2004), supporting a mechano-sensory role for NO, stabilizing the cytoskeleton via its interaction with talin and vinculin. In the heart, the loss of talin-1 and talin-2 is associated with disrupted expression of β1-integrin and associated proteins at the cardiomyocyte membrane, and promotes cardiomyocyte dysfunction leading to cardiomyopathy (Manso et al., 2017). Vinculin co-localizes at the costameres and intercalated discs of cardiomyocytes (Zemljic-Harpf et al., 2009) and its loss is associated with impaired cardiomyocyte organization (Zemljic-Harpf et al., 2007). These findings implicate the critical roles of talin and vinculin in integrin activation and maintenance of cardiomyocyte membrane stability. However, the impact of NO signaling on cardiac talin and vinculin remains to be investigated. The effect of NO on the integrin system are summarized in Figure 1.

### Dystrophin and the Dystrophin Glycoprotein Complex

**FIGURE 2:**
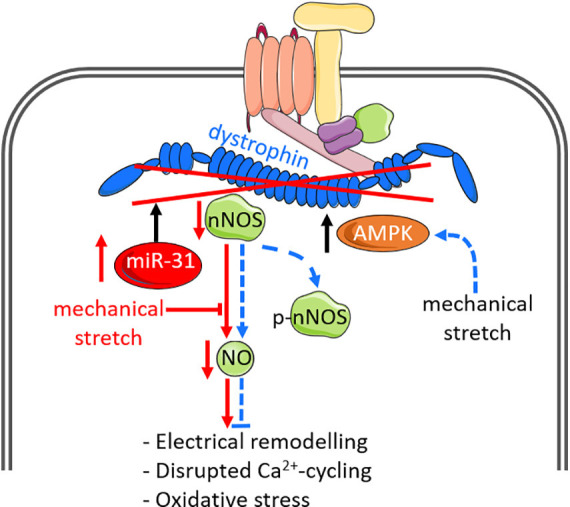
Nitric oxide signaling and mechanical stimuli affect dystro-sarcoglycan complex function and can modify disease pathology. Dystrophin is the largest component of the dystrophin-glycoprotein complex (DGC), and closely associates with neuronal nitric oxide synthase (nNOS). Loss of dystrophin, via upregulation of microRNA-31 or genetic mutations, leads to disassociation of nNOS from the DGC and impairs NO production. Loss of NO is associated with atrial electrical remodeling, disrupted Ca^2+^ cycling and oxidative stress. The DGC additionally acts as a mechano-sensor to regulate NO signaling. In normal cardiomyocytes in which dystrophin is intact (indicated by dashed blue lines), mechanical stretch enhances NO production via enhanced AMP-activated protein kinase (AMPK) activation and phosphorylation of nNOS-serine 1412 (p-nNOS), which increases nNOS activity. Such downstream effects by mechanical stretch are impaired in dystrophin-deficient cardiomyocytes. The impact of dystrophin-deficiency on NO production are indicated by solid red lines.

The DGC is a network of several membrane-associated proteins that provides structural integrity to the plasma membrane and facilitates transduction of mechanical forces from the sarcomere and intracellular cytoskeleton to the extracellular matrix during contraction (Lapidos et al., 2004). As an important component of this cytoskeletal complex, dystrophin is closely associated with nNOS (Figure 2), and loss of dystrophin has adverse consequences on the localization and regulation of nNOS (Brenman et al., 1995). In skeletal muscle, loss of dystrophin uncouples nNOS from the sarcolemma to the cytosol and depletes NO synthesis by > 70% (Brenman et al., 1995), and promotes skeletal muscle pathophysiology (Thomas et al., 2003; Percival et al., 2010; Froehner et al., 2015). In the heart, dystrophin is localized to the cardiomyocyte sarcolemmal membrane and T-tubules (Kaprielian et al., 2000; Garbincius and Michele, 2015), whilst nNOS is localized to the sarcoplasmic reticulum and sarcolemmal membrane (Xu et al., 1999; Oceandy et al., 2007; Carnicer et al., 2017). Loss of dystrophin at the sarcolemmal membrane thus displaces nNOS from the sarcolemmal membrane and contributes to altered nNOS regulation. This dysregulation also disrupts the Ca^2+^-cycling machinery in cardiomyocytes (Sears et al., 2003; Zhang et al., 2008), leading to impaired contractile function. Indeed, atrial dystrophin protein is repressed by atrial-specific upregulation of micro-RNA-31 (miR-31) in humans and goats with atrial fibrillation (Reilly et al., 2016). Reduced dystrophin protein disrupts the protein stability of nNOS and impairs NO synthesis, which promotes atrial electrical remodeling including shortening of action potential duration and increased repolarizing K^+^ currents (Reilly et al., 2016). These findings suggest important roles for dystrophin and NO signaling in cardiac electrophysiology and function. A study in dystrophin-deficient *mdx* mice previously reported associations between reduced nNOS activity or expression and cardiac ECG abnormalities (Bia et al., 1999). Similarly, transgenic overexpression of nNOS in aged *mdx* mice mitigated myocardial fibrosis and inflammation, cardiac ECG abnormalities and premature ventricular contractions (Wehling-Henricks et al., 2005). Enhancing NO signaling in response to impaired components of the DGC may be protective against mechanical stress. A study on dystrophin/utrophin-deficient cardiomyocytes used whole body periodic acceleration (a technique that induces pulsatile shear stress to the endothelium, by sinusoidal head to foot motion of the supine body) to increase endogenous expression of eNOS and nNOS (Adams et al., 2009). They showed that increased NO production ameliorated impaired cardiomyocyte contractile function, oxidative stress and elevated intracellular diastolic Ca^2+^ and Na^+^ concentrations that were induced by mechanical stress (Lopez et al., 2017).

The DGC also functions as a mechano-sensor to directly regulate muscle NO signaling. In dystrophin-deficient *mdx* cardiomyocytes, mechanical stretch impairs cardiac NO production and is associated with impaired phosphorylation of nNOS-serine 1412 (Garbincius and Michele, 2015), a phosphorylated site known to enhance nNOS activity (Rameau et al., 2007), implicating the direct effects of dystrophin on mechanically activated NO signaling. Further investigations revealed that mechanical stretch promotes phosphorylation of nNOS by AMP-activated protein kinase (AMPK) activation, which is impaired in *mdx* cardiomyocytes, suggesting dystrophin is necessary for mechanically regulating AMPK and hence NO production (Garbincius and Michele, 2015). These findings suggest that the DGC and dystrophin not only serve as a passive scaffold for nNOS to bind on, but they also exert direct effects on NO production via AMPK activation and subsequent posttranslational modifications of nNOS.

### LIM Domain Proteins

In addition to the integrin complex and DGC, there are other cytoskeletal proteins that exhibit mechanical sensing properties that are influenced by NO signaling. LIM domain proteins are a family of transcriptional and cytoskeletal proteins characterized by their LIM domains containing a double-zinc finger motif with protein-binding interfaces (Kadrmas and Beckerle, 2004). These proteins exhibit a diverse range of biological roles, including mechano-transduction (Smith et al., 2014). The LIM domain family proteins dynamically regulate the remodeling and repair of actin stress fibers, which reinforces the cytoskeleton in response to mechanical forces (Yoshigi et al., 2005; Smith et al., 2013, 2014). Two key LIM domain proteins that exhibit mechano-sensory roles and are influenced by NO signaling are Muscle LIM Protein (MLP) and Lipoma Preferred Partner (LPP). Whilst they are not structural components of the sarcomere or the cardiac cytoskeleton, both proteins transiently associate with these structures as part of their signaling function. Muscle Lim Protein (MLP) is a key nucleocytoplasmic shuttling protein located on the sarcomeric Z-disc that exhibits cardiac mechanical sensing properties (Knoll et al., 2002). The protein exhibits pleiotropic biological processes in the maintenance of cytoskeletal and sarcomere function including actin polymerization/depolymerization (Arber et al., 1997; Gupta et al., 2008; Papalouka et al., 2009). Deficiency of MLP is associated with misalignment of the Z-disc, loss of passive stretch sensing, and disruption of an interaction between MLP, telethonin, and titin, which forms a mechano-sensing complex at the Z-disc (Knoll et al., 2002). One of the roles of MLP is to inhibit protein kinase C-α (PKCα) at the Z disk, and loss of MLP can result in heart failure through removal of these inhibitory actions (Lange et al., 2016). Interestingly, NO exerts antihypertrophic effects by promoting transcriptional and translational downregulation of MLP via cGMP-dependent activation of PKG (Heineke et al., 2003), and inhibition of NO prevented upregulated MLP protein and sarcomere disarray (Hooper et al., 2013). Further, subcellular fractionation experiments revealed re-localization of MLP from the cytosol to the nucleus following inhibition of NO by L-NAME treatment (Hooper et al., 2013), in which nuclear MLP has been demonstrated to promote hypertrophic growth in response to mechanical stress (Boateng et al., 2009).

Lipoma-preferred partner (LPP) is another nucleocytoplasmic shuttling protein localized on focal adhesions and cell–cell junctions, and communicates between the nucleus and cytoskeleton (Petit et al., 2000). It has also been described as a cardiac mechano-sensitive protein highly expressed in cardiac fibroblasts and participates in the regulation of cellular adaptations in response to cardiac hypertrophy induced by hemodynamic overload (Hooper et al., 2012). LPP also associates with the actin cytoskeleton to inhibit actin polymerization (Hansen and Beckerle, 2006). Indeed, it is proposed that NO regulates LPP expression following mechanical stress.

Treatment of cardiac fibroblasts with L-NAME and subjected to 5% stretch rescued downregulated LPP protein levels induced by stretch (Hooper et al., 2012), suggesting that stretch-induced downregulation of LPP is dependent on NO production. In cardiomyocytes, mechanical stress may produce NO and promote subcellular localization of LPP that inhibits myocyte growth. Treatment of cardiomyocytes with L-NAME under control and with 10% stretch decreases nuclear and cytoskeletal expression of LPP protein levels (Hooper et al., 2013). Nuclear LPP represses of transcription factors related to cellular growth (Guo et al., 2006b), whilst cytoskeletal LPP limits the rate of actin polymerization by inhibiting vasodilator stimulator phosphoprotein activity (Bear et al., 2002). Thus, mechanically regulated NO may regulate cellular growth and actin polymerization in cardiomyocytes by altering the subcellular localization of LPP. Collectively, these findings highlight the significant impact NO production and NO-derived signaling exerts on the mechano-sensing properties of MLP and LPP.

**FIGURE 3:**
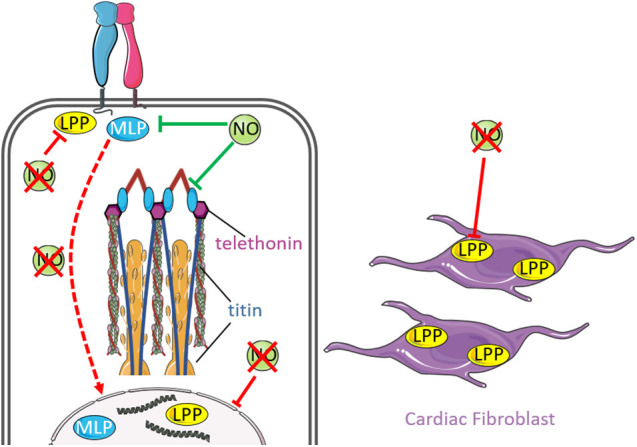
Nitric oxide signaling and mechanical stimuli affects signaling by MLP and LPP. Muscle LIM protein (MLP) is a nucleocytoplasmic protein that interacts with integrins at costameres and forms a mechano-sensing complex with titin and telethonin at the sarcomeric Z-disc (inset). Interestingly, enhanced NO downregulates MLP expression, which is associated with disruption of the MLP-titin-telethonin complex and misalignment of the Z-disc. Loss of NO promotes re-localization of MLP from the cytosol to the nucleus (red dashed arrow), where nuclear MLP has been demonstrated to promote hypertrophy in response to mechanical stress. Lipoma preferred partner (LPP) is another nucleo-cytoplsmic protein that is localized at focal adhesions, cell-cell junctions and communicates between the nucleus and cytoskeleton. The protein is highly expressed in cardiac fibroblasts and participates in cellular adaptations in response to mechanically induced cardiac hypertrophy. Nitric oxide has been proposed to regulates LPP expression following mechanical stress. Loss of NO is associated with reduced expression of nuclear and cytoskeletal LPP expression.

Figure 3 summarizes the effect of NO and mechanical stress on MLP/LPP signaling.

### Titin

Titin is the largest protein in the sarcomere, forming the third filament system and is hence an important part of the cardiac cytoskeleton. It acts as a molecular “spring.” One titin molecule spans half a sarcomere from the Z-disc to the M-band (Linke, 2018). Whilst it is not actively involved in mechanical contractility, titin is the biggest contributor to elasticity and stretch activation (Linke, 2018). Titin is a mediator for mechano-sensory processes due to its ability to form multiple protein-protein interactions throughout the sarcomere (Kruger and Linke, 2009). The function of titin on passive tension is mediated by cyclic GMP-dependent PKG-mediated phosphorylation of several sites within the spring region of titin (Kruger et al., 2009; Leite-Moreira et al., 2018). PKG-mediated phosphorylation of titin at serine residue S469 alters the elasticity of N2B-unique sequence of titin, leading to reduced passive tension in human donor non-failing cardiac fibers (Kruger et al., 2009). Acute stretch of cardiomyocytes decreased passive tension, elevated PKG activity and phosphorylation of titin; the latter was dampened in acutely stretched cardiomyocytes that were pre-incubated with inhibition of PKG (Leite-Moreira et al., 2018). These observations further support a role of PKG in mediating phosphorylation of titin in response to stretch. Inhibition of NO, natriuretic peptide and NO scavenging in acutely stretched rabbit cardiomyocytes reduced passive tension decay that was similarly observed by inhibition of PKG only, suggesting that activation of cGMP-dependent PKG-mediated phosphorylation of titin may be partly mediated by the synergistic effects of NO and natriuretic peptide (Leite-Moreira et al., 2018). Other posttranslational modifications on titin, including S-glutathionylation (Alegre-Cebollada et al., 2014) and S-nitrosylation (Figueiredo-Freitas et al., 2015), also contribute to alterations in titin elasticity, supporting a role for NO signaling in regulating the elasticity of titin.

## Ion Channel Modulation by Mechanical Stress

Contraction of the heart is initiated in response to the electrical depolarization of the tissue. Tissue depolarization occurs in the form of a wave of action potentials, originating in the sino-atrial node. However, the relationship between electrical signal and mechanical response is more nuanced than this simplistic view. The term “mechano-electrical coupling” has been coined to describe the dynamics of electrical and mechanical feedback in the context of cardiac function. Ion channels are well placed to integrate changes in the mechanical environment into an altered electrical response: sitting at the junction of cell membrane and signal transduction they are exquisitely placed to transform a mechanical signal into a biological response. Therefore, gating of ion channels in response to mechanical stimuli, results in changes to the ionic composition of the cell, which can act as a biological signal to drive responses to the change in mechanical environment. Numerous ion channels have been shown to be either directly gated by changes to the mechanical environment or mechanically sensitive (i.e., their function is modified by mechanical forces in an indirect manner through changes to the cytoskeleton or secondary messengers). The extensive number of cardiac ion channels regulated by mechanical stimuli has been excellently reviewed by Peyronnet and colleagues (Teng et al., 2014; Peyronnet et al., 2016). In the heart there are two main currents known to be activated in response to stretch. These are a stretch activated potassium current *I*_*SA*__*C,K*_ and the non-selective cation conductance G_*ns*_. I_*SA*__*C,K*_ is likely to be composed of a K2P channel such as TREK or TRAAK, a K_*ATP*_ channel, a BK channel (Reed et al., 2014) with contributions from the other many mechanically sensitive potassium channels (see Table 1). The molecular identity of G_*ns*_ has not been categorically determined, but may be a TRP channel or Piezo 1 (reviewed in Reed et al., 2014; Peyronnet et al., 2016). Whilst there are many known mechanically sensitive ion channels which participate in the mechano-transduction process, many of these are classical voltage gated ion channels (which are generally considered to be gated by changes to the membrane potential) which are also able to be modulated by mechanical stimuli (Peyronnet et al., 2016). In order to be considered mechanically *gated*, three criteria have been proposed (Ridone et al., 2019). These are:

**TABLE 1:** Cardiac ion channels modulation by nitric oxide and mechanical stress.

**Channel**	**Mechanical response**	**Affected by NO**
**Potassium**		
K_*v*_1.5 (*I*_*Kur*_)	Increased at membrane in response to shear stress	Inhibited (Nunez et al., 2006; Al-Owais et al., 2017)
K_*v*_7.1 (*I*_*Ks*_)	Increased in response to hypotonic stretch (Calloe et al., 2007)	Increased (Asada et al., 2009)
hERG (*I*_*Kr*_)	Increased by shear stress (Roy and Mathew, 2018)	Inhibits (Taglialatela et al., 1999)
K_*v*_6.2 (K_*ATP*_)	Increased by hypotonic stretch (Van Wagoner, 1993)	Increased (Cameron et al., 2003)
K2P	Increased by stretch of cell membrane via patch pipette (TREK-1) (Bagriantsev et al., 2011)	Increased (TALK-1) (Lu et al., 2007) Increased (TREK-1) (Koh et al., 2001)
K_*ir*_ (*I*_*K1*_)	Increased in response to stretch (Dyachenko et al., 2009)	Increased (Gomez et al., 2009)
BK channels	Increased by stretch (Zhao et al., 2010)	Increased (Kyle et al., 2017)
**Sodium**		
Na_*v*_1.5 (*I*_*Na*_)	Peak increased (Beyder et al., 2010) in response to stretch of cell membrane via patch pipette (Beyder et al., 2010)	Peak decreased, late increased (Ahern et al., 2000)
**Calcium**		
Ca_*v*_1.2 (*I*_*Ca,L*_)	Increased in response to stretch (Takahashi et al., 2019) Stability at the membrane increased (Pedrozo et al., 2015)	Inhibited (Caballero et al., 2010)
**Non-selective**		
TRPC6	Increased in response to cell stretching (Nikolova-Krstevski et al., 2017)	Inhibited (Seo et al., 2014)
Piezo-1	Increased by stretch (Coste et al., 2010)	Activation of the channel may lead to increased NO production (Song et al., 2019)
**Connexins**		
Connexin 43	Expression increased in response to stretch (leading to increased conduction velocity) (Zhuang et al., 2000; Meens et al., 2013)	Permeability increased in response to NO via sGC (Crassous et al., 2017)

1. Removal of the ion channel abolishes the mechano-sensory response of the cell.
2. The mechano-sensory response is disrupted if key structural residues are altered in the ion channel.
3. Over expression of the ion channel in a non-mechano-sensory cell renders it mechano-sensory.

In mammalian systems, the complexity and biological redundancy associated with mechano-transduction processes mean that fulfilment of all these criteria is difficult to prove. However, candidate ion channels which do meet these requirements are the Piezo 1 and 2 channels (see below).

Mechanically sensitive (rather than gated directly by mechanical stress) ion channels also pass current in response to changes in the mechanical environment. However, the mechanisms involved in their gating are diverse and may involve indirect processes. Some examples of the different ways in which mechanically sensitive ion channels gate are change in subcellular localization (Boycott et al., 2013) and change in stability of the channel at the membrane and changes to membrane tension (Honore, 2007; Pedrozo et al., 2015). There is also evidence that the cytoskeleton is a key regulator of ion channel mechano-sensitivity (Martinac, 2014), indeed the regulation of K_*v*_1.5 by shear stress requires an intact microtubule network (Boycott et al., 2013).

The functional properties of some ion channels can be modified by NO, which can occur via the addition of a nitroso group to a thiol (Gonzalez et al., 2009), for example, on a cysteine residue. Such nitrosylation can have the effect of enhancing the channel activity or inhibiting it depending on the specific ion channel involved. In particular there are several channels which are both mechanically sensitive and modulated by NO, such as voltage dependent potassium channel-1.5 (K_*v*_1.5) (Nunez et al., 2006), voltage dependent calcium channel-1.2 (Ca_*v*_1.2) (Campbell et al., 1996) and voltage dependent sodium channel-1.5 (Na_*v*_1.5) (Ueda et al., 2008). NO also affects the transcriptional regulation of some ion channels via activation of the cGMP/PKG pathway. Certain ion channels, e.g., K_*v*_1.5 can be modulated by NO through both direct nitrosylation and the PKG pathway (Nunez et al., 2006). This review will not evaluate the effects of NO on cardiac electrophysiology, as these have been excellently reviewed elsewhere (Tamargo et al., 2010), but will instead focus on the ion channels known to be affected by NO and mechanical stress, and the link between them.

**FIGURE 4:**
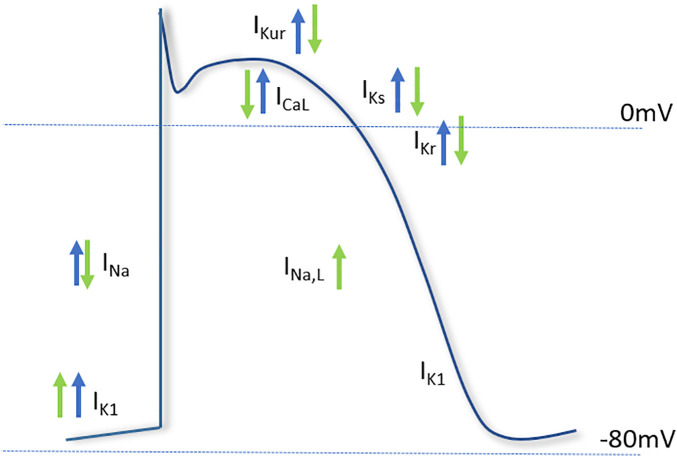
Major cardiac ion channels known to be affected by nitric oxide and mechanical stimuli. The effect of nitric oxide (green arrows) and mechanical stress (blue arrows) on the major currents of the cardiac action potential. The depolarizing sodium current (I_*Na*_), responsible for the upstroke of the action potential, is increased by mechanical stress and decreased by NO. The late component of I_*Na*_ is increased by NO. The L-type calcium current (I_*CaL*_), involved in the plateau phase of the action potential, is inhibited by NO, but increased by mechanical stress. Of the repolarizing potassium currents, NO inhibits the ultrarapid delayed rectifier current (I_*Kur*_), the rapid delayed rectifier (I_*Kr*_) and slow delayed rectifier (I_*Ks*_), whereas the inwardly rectifying potassium current (I_*K*__1_) is increased by NO. Mechanical stress has been shown to activate the repolarizing currents I_*Kur*_, I_*Ks*_, I_*Kr*_, and I_*K*__1_.

There is some evidence that NO is released in response to mechanical stimuli (Petroff et al., 2001; Shim et al., 2017a), however, reports investigating the relationship between NO and mechano-electrical conduction (through modulation of ion channel activity) have been somewhat limited. At the level of the action potential, it has been reported that stretch of rat sino-atrial node cardiomyocytes prolongs the action potential duration (APD), and leads to fibrillation. The authors also found that the exogenous application of NO using the donor molecule S-Nitroso-N-acetylpenicillamine (SNAP) exacerbated the fibrillation, and prevented a return to control APD upon cessation of stretch. The authors conclude that increased NO during stretch is responsible for the opening of mechanically gated ion channels, and that in the presence of the NO donor this led to unresolvable fibrillation. The ionic currents underlying the findings were not described, and the role of endogenous NO in the response to stretch was not tested (Shim et al., 2017b). The major ionic currents known to be modulated by NO and mechanical stress and their potential contribution to the action potential is described in Figure 4.

The following section explores the evidence for regulation of specific cardiac ionic currents by mechanical stimuli, and the potential role for NO in this regulation.

### The L-Type Calcium Channel

The L-type calcium current (LTCC) mediated by Ca_*v*_1.2 is involved in action potential prolongation, maintaining phase two of the cardiac action potential. The influx of Ca^2+^ into cardiomyocytes following depolarization activates ryanodine receptors and leads to sarcoplasmic reticulum Ca^2+^ release and ultimately to cardiac contraction. As a result, the LTCC is critical for excitation-contraction coupling, linking the electrical signal of the action potential to the mechanical response of the cardiomyocyte manifested as contraction. A recent study demonstrated that the LTCC is involved in Ca^2+^ release in response to the stretch of a rat cardiomyocyte cell line (Takahashi et al., 2019). The channel is also affected by NO, which inhibits the channel. Ca^2+^ currents from mice lacking nNOS are larger and inactivate more slowly than wild type littermates, suggesting that NO tonically inhibits the channel (Caballero et al., 2010). Lack of nNOS is arrhythmogenic in mice, partially as a result of the removal of inhibition on the LTCC (Sears et al., 2003; Burger et al., 2009). Interestingly, nitrosylation of Ca_*v*_1.2 is increased in rats in response to mechanical stress induced by isoproterenol treatment (Kentish et al., 2001; Sun et al., 2006). The nitrosylation is protective against ischemia-reperfusion injury, consistent with nitrosylation inhibiting the LTCC (Sun et al., 2006).

### Sodium Current

The main depolarizing current in cardiomyocytes is mediated through the inward flow of Na^+^ ions, carried by the ion channel Na_*v*_1.5. The influx of Na^+^ into the cell initiates the depolarization necessary to reach the threshold for action potential generation. In cardiomyocytes, Na_*v*_1.5 forms a complex with nNOS and syntrophin at the sarcolemma (Ueda et al., 2008). Direct nitrosylation of Na_*v*_1.5 has been demonstrated in several studies and exerts a dual effect on the channel. Firstly, the peak current is decreased, secondly, the so-called late persistent current (which does not inactivate) is increased (Ahern et al., 2000). Na_*v*_1.5 is mechano-sensitive, with stretch increasing the peak current and accelerating inactivation (Peyronnet et al., 2016). In addition, Chorro et al. (2015) demonstrated that ranolazine (which selectively inhibits the late sodium current) application to Langendorff perfused rabbit hearts reduced stretch-induced ventricular fibrillation. The same group also showed that a more selective blocker of late inward sodium current (I_*Na,L*_) (GS967) was similarly capable of preventing the stretch induced effects on cardiac refractoriness (Del Canto et al., 2018). Although the effect of NO in this process was not assessed it is conceivable that the stretch induced increase in late Na^+^ current could be mediated by NO.

### TRPC6

Transient Receptor Potential C6 (TRPC6) channels are non-voltage gated cation channels. They are potentially involved in the atrial response to stretch, as endothelial TRPC6 changes its localization, expression and activity in response to stretch. This results in paracrine effects on cardiomyocytes which alters Ca^2+^ signaling (Nikolova-Krstevski et al., 2017). The authors suggest that the channel is directly modulated by stretch, as GsMTx-4 which disrupts TRPC6 interaction with the surrounding membrane lipids and can prevent the resultant effects of stretch on channel activity. Similarly, stretch of murine ventricular myocytes causes an increase in G_*ns*_, which may be a result of TRPC6 activation (Dyachenko et al., 2009). The stretch induced increase in G_*ns*_ is dependent on the action of NO, generated by eNOS. The authors found that the effect of stretch also required superoxide, and conclude that peroxynitrite is the stretch dependent signaling molecule responsible for the increase in G_*ns*_. The TRPC6 channel can be inhibited by the PKG-pathway, and this regulation is necessary for the increase in Ca^2+^ seen upon stretch of myocytes with carbon-fiber rods (Seo et al., 2014).

### Piezo-1

Discovered in 2010, Piezo-1 is a non-selective cation channel, which is directly gated by mechanical forces (Coste et al., 2010). Although the expression of Piezo-1 does not seem to be high in cardiomyocytes, there are intriguing hints that the protein may have a role to play in cardiomyocyte mechano-transduction. Piezo-1 is expressed in rodent myocardium, and is upregulated in response to heart failure and angiotensin II treatment (Liang et al., 2017). The protein is also expressed in human cardiac fibroblasts where it induces release of interleukin-6 (IL-6) (Blythe et al., 2019). In the endothelium, Piezo-1 mediates NO production in response to shear stress (Wang et al., 2016). The human cardiac cell line AC16 expresses Piezo-1 which is downregulated in response to prolonged stretching, and may modulate the phosphorylation (and hence activity) of eNOS (Wong et al., 2018). Interestingly, in drosophila neurons, Piezo-1 activation leads to an increase in intracellular Ca^2+^ which results in an increase in NO production and stimulation of the PKG pathway (Song et al., 2019). The extent to which Piezo-1 contributes to cardiac mechano-electrical signaling, and the involvement of NO remains to be explored. If Piezo-1 affects cardiac NO production it may be that the channel is important for the increase in NO production observed in response to mechanical stress, thus placing Piezo-1 at the start of the NO mediated mechanical signaling cascade.

### Potassium Channels

Direct evidence linking potassium channels, NO and mechanical responsiveness is lacking at the present time. However, there are several channels which are both mechanically sensitive and modulated by NO, raising the possibility that a link may be present.

Interestingly, our group has shown that atrial cardiomyocytes taken from patients in chronic atrial fibrillation (a condition in which the mechanical environment is likely to be perturbed, in which nNOS is depleted, and the potassium currents *I*_*Kur*_ and *I*_*K*__1_ are increased) leads to the electrical remodeling characteristic of the condition (Reilly et al., 2016). Further research is necessary to understand the role of NO in the mechanical regulation of cardiac K^+^ currents in health and disease. The use of specific NOS inhibitors to examine the effect of mechanical stress on K^+^ currents could be a useful tool to improve understanding of the role of NO in mediating the mechanical response of these channels.

## Calcium Handling and Homeostasis

Precise regulation of intracellular Ca^2+^ levels are fundamental for the proper contractile response of cardiomyocytes. Within cardiac myocytes, Ca^2+^ signaling is tightly controlled in dedicated microdomains, myocyte mishandling of Ca^2+^ can cause both contractile dysfunction and arrhythmias in pathophysiological conditions (Schonleitner et al., 2017). During an action potential, Ca^2+^ enters the cell through the L-type Ca^2+^ channel, located in t-tubular membrane invaginations, during depolarization. The influx of a small amount of Ca^2+^ activates the underlying ryanodine receptors (RyR) located on the adjacent sarcoplasmic reticulum (SR) membrane. Electron microscopy and high resolution immunofluorescence images show that t-tubular L-type Ca^2+^ channels and RyR form regular and co-localized patterning within 10–12 nanometers in structures known as dyads (Page and Buecker, 1981; Koh et al., 2006). Ca^2+^ binding to the cytoplasmic extremity of the RyR channel pore triggers increased opening probability and concurrent release of Ca^2+^ from the SR via Ca^2+^ induced Ca^2+^ release (CICR) which amplifies the intracellular Ca^2+^ concentration 5–10-fold (Bers, 2002; del Rio et al., 2009; Schonleitner et al., 2017). Cytoplasmic Ca^2+^ influx from the SR is detected primarily by sarcomeric troponin C (TnC) on the actin filament, Ca^2+^ binding to the regulatory site II EF hand motif of TnC causes the release of the inhibitory peptide of troponin I from actin, and along with troponin T and tropomyosin activates actomyosin S1 ATPase dependent contraction of the cardiomyocyte (Tobacman, 1996; Bers, 2002). RyR located on the SR membrane mediate the release of Ca^2+^ from the SR when they are activated by the binding of Ca^2+^, meaning that RyR are central to Ca^2+^ amplification during CICR (Bers, 2002; Jian et al., 2014). For relaxation to occur, Ca^2+^ must be released by TnC in diastole and removed from the cytoplasm. This requires closure of the RyRs and Ca^2+^ to be pumped (1) back into the SR, by the sarco-endoplasmic reticulum Ca^2+^ ATPase (SERCA) and (2) out of the cell, largely by the sodium–calcium exchanger (NCX) and sarcolemmal Ca^2+^ ATPase (Eisner et al., 2017). The SERCA pump is regulated by the small transmembrane protein phospholamban (PLN) which acts as a molecular break and binds to SERCA when dephosphorylated, upon phosphorylation by either protein kinase A (PKA) or calcium-calmodulin Ca^2+^/calmodulin-dependent protein kinase II (CaMKII), PLN dissociates form SERCA and pentamerizes (Kranias and Hajjar, 2012). This process along with phosphorylation of key sarcomeric proteins and Ca^2+^ handling proteins such as troponin I, myosin regulatory light chain, RyR, and L-type Ca^2+^ channel forms the key processes that regulate the physiological fight or flight response in the heart in response to β-adrenergic stimulation (Bers, 2002; Najafi et al., 2016). The processes by which cardiomyocytes sense mechanical load and respond by changing Ca^2+^ dynamics have been termed mechano-chemo-transduction (MCT). Mechano-chemo-transduction pathways provide autoregulation of Ca^2+^ signaling and contractility in normal hearts, but may cause Ca^2+^ dysregulation in diseased hearts (Chen-Izu and Izu, 2017).

### SR Ca^2+^ Release

Although Ca^2+^ release from the SR is usually triggered by opening of the L-type calcium channel, changes to RyR sensitivity may result in the localized spontaneous release of Ca^2+^ in a so-called Ca^2+^ spark. Under certain conditions, such as a high SR Ca^2+^ content, or increased RyR sensitization (such as channel phosphorylation by PKA or CAMKII), the Ca^2+^ spark can propagate to neighboring RyR clusters, and an arrhythmic Ca^2+^ wave is triggered (Schonleitner et al., 2017). Stretch-induced increases in Ca^2+^ spark frequency have been consistently observed in myocytes (Prosser et al., 2011), and are also found in response to shear stress and afterload (Jian et al., 2014). The existence of stretch induced Ca^2+^ sparks demonstrated that the Frank-Starling effect, previously thought to be Ca^2+^ independent, involves fast, local changes in Ca^2+^. These are believed to be a result of changes to the sensitivity of SR Ca^2+^ release channels (Schonleitner et al., 2017). Excessive mechanical loading and stress can cause a high rate of diastolic Ca^2+^ sparks, which are able to trigger spontaneous Ca^2+^ waves leading to arrhythmic events (Katra and Laurita, 2005; Jian et al., 2014).

RyR sensitivity can be affected by nitrosylation, which increases the activity of the channel (Wang et al., 2010; Johnson and Antoons, 2018). nNOS is localized between the SR and sarcolemma where it associates with various partner proteins, including SERCA and RyR. This proximity results in the modulation of RyR activity and SERCA by nNOS and increase in Ca^2+^ sparks and Ca^2+^ reuptake (Jian et al., 2014). nNOS increases RyR Ca^2+^ leak either directly via S-nitrosylation (Johnson and Antoons, 2018) or indirectly via CaMKII (Jian et al., 2014; Erickson et al., 2015). nNOS also facilitates SERCA Ca^2+^ reuptake (Vielma et al., 2016), which may compensate for increased SR Ca^2+^ leak (Carnicer et al., 2017). Redistribution of nNOS in the failing heart has been reported, and is thought to contribute to the pathology. This is potentially a consequence of the loss of micro domain structures seen in many pathological conditions (Schonleitner et al., 2017).

How the cell senses the mechanical stress stimulus and how the stimulus is transmitted to intracellular Ca^2+^ stores remain controversial. The study by Jian et al. (2014) demonstrated a relationship between mechanical load and NO production. Using a cell-in-gel system to recreate the forces experienced in the native cardiomyocyte, the authors showed that load mediated Ca^2+^ dynamics are reliant on NOS enzymes. The study also suggested differential effects for nNOS and eNOS in modulating different Ca^2+^ handling pathways. Both NOS isoforms are involved in elevating Ca^2+^ transients; however, afterload-induced spontaneous Ca^2+^ sparks are mediated by nNOS, but not eNOS. The explanation for the divergent effects of the NOS isoforms may be due to their different subcellular localizations (Jian et al., 2014).

In these settings, NOS produces NO by phosphorylation via the phosphoinositide 3-kinase/protein kinase B (PI3K-Akt) pathway (Petroff et al., 2001). nNOS is also centrally involved in stretch induced Ca^2+^ spark production, most likely via oxidation of CaMKII (Gutierrez et al., 2013; Jian et al., 2014) which increases RyR activity and SR Ca^2+^ load. Increased phosphorylation of RyR by CaMKII has been proposed to be a feature of hypertrophic cardiomyopathy genetic mutations (Robinson et al., 2018). Interestingly overexpression of iNOS specifically in cardiomyocytes of transgenic mice results in cardiac hypertrophy and sudden cardiac death (Mungrue et al., 2002), whilst energetic depletion as a result of pathogenic cardiomyopathy mutations results in increased the generation of reactive oxygen species by the mitochondria (Ashrafian et al., 2003; Sacchetto et al., 2019). The specific role of other NOS isoforms in this pathology is unknown.

### CaMKII

**FIGURE 5:**
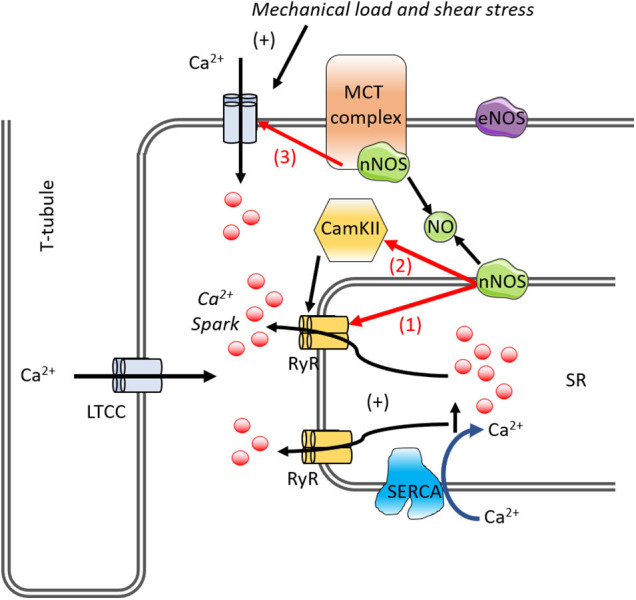
A diagram of the mechano-chemo transduction pathways in the cardiomyocyte. Mechano-transduction involves NO for RyR activation. In cardiomyocytes, mechanical load, and shear stress causes Ca^2+^ influx, Ca^2+^ binds to and activates the underlying RyR. This increases spontaneous Ca^2+^ spark via RyR. nNOS and CaMKII is activated promoting SR Ca^2+^ leak via RyR phosphorylation. nNOS increases RyR Ca^2+^ leak either (1) directly via S-nitrosylation or (2) indirectly via CaMKII. The (3) LTCC channel is affected by NO, inhibiting the channel. LTCC, L-type calcium current; NO, nitric oxide; nNOS, neuronal nitric oxide synthase; RyR, ryanodine receptor; SERCA, SR Ca^2+^-ATPase; SR, sarcoplasmic reticulum; CaMKII, Ca^2+^-calmodulin-dependent protein kinase II.

Downstream of NOS signaling, CaMKII has been found to regulate Ca^2+^ homeostasis and cardiac function and also mediate the MCT effect on Ca^2+^ handling (Jian et al., 2014; Chen-Izu and Izu, 2017). CaMKII can phosphorylate several Ca^2+^-handling proteins including phospholamban, RyR, and the L-type Ca^2+^ channel, as well as myofilament proteins to stimulate Ca^2+^ sparks (Hidalgo et al., 2013; Jian et al., 2014). Recent reports have implied that NO can activate CaMKII in the heart (Erickson et al., 2011; Jian et al., 2014). Mechanical stress activates NOS which, through NO and ROS signaling, modulate CaMKII activity. These signaling pathways orchestrate the modulations of Ca^2+^ handling molecules to regulate Ca^2+^ dynamics in response to mechanical stress (Chen-Izu and Izu, 2017). Phosphorylation of the cardiac RyR is suggested to be important not only for normal cardiac excitation-contraction coupling (Marx et al., 2000; Wehrens et al., 2004) but also in exacerbating abnormalities in Ca^2+^ homeostasis that are seen in heart failure (Marx et al., 2000; Belevych et al., 2007) and cardiomyopathies (Robinson et al., 2018). It is known that CaMKII-dependent phosphorylation of RyR can give rise to an increase in open probability (Marx et al., 2000; Guo et al., 2006a). CaMKII contributes to afterload-induced Ca^2+^ sparks (Guo et al., 2006a) and the inhibition of both nNOS and CaMKII in cardiomyocytes eliminates the Ca^2+^ sparks, suggesting mechano-transduction activates nNOS and CaMKII (Jian et al., 2014). Mechano-chemo-transduction through NOS and CaMKII signaling pathways suggests possible therapeutic targets for treating mechanical stress-induced Ca^2+^ dysregulation, arrhythmias (Jian et al., 2014), and cardiomyopathy (Moolman et al., 1997; Alvarez et al., 1999). The effects of NO in mediating Ca^2+^ handling in the cardiomyocyte are shown in Figure 5.

## Discussion

The ability of the heart to respond appropriately to changes in the mechanical environment is important for proper cardiac function. Production of NO by nNOS and eNOS regulates every stage of mechano-electrical transduction; from initial sensing of mechanical signals through the integrin/cytoskeleton complexes, via control of the action potential by modulation of ion channel activity, through to mediating the calcium handling processes underpinning cardiac contraction.

Dysfunction of these processes in pathological conditions make understanding the complex role of NO vital in order to yield effective therapeutics based on NO replacement. It is clear that we still do not fully understand the way in which NO derived from mechanical signaling alters mechano-electrical signaling, and more work is needed, particularly in understanding how ionic currents are altered, and how this may result in arrhythmogenic activity. As technological advances occur it is becoming more feasible to investigate the contributions of different types of mechanical stresses to cardiac physiology. For example, acrylamide gels which allow approximation of native physiological and pathophysiological stiffness are used to investigate cardiomyocyte response to different surfaces (Pandey et al., 2018). A 3D cell-in-gel system allows the effect of preload to be investigated when the cardiomyocyte contracts again the gel (Shaw et al., 2013). Alternatively, myocytes can be adhered to stiff glass rods using Myotak^TM^ to allow them to be stretched along the long axis (Prosser et al., 2011; Peyronnet et al., 2017). Similarly, force transducers and length controllers previously used to study chemically skinned cardiac trabeculae (Reiser et al., 1994; Simnett et al., 1994; Robinson et al., 2002), are now beginning to be used to study physiologically intact muscle strips in both cardiac and skeletal muscle (Tomalka et al., 2019). Magnetic tweezers can also be used to allow precise control of the strength and direction of force on myocytes (Chen et al., 2016). Finally, microdomain effects of stretch have been investigated using hydrojets from a micropipette to interrogate a highly specific area of the cell membrane, the jet causes indentation of the membrane in a similar way to atomic force microscopy, however, the area covered is more locally defined (Sanchez et al., 2008). These techniques have also been reviewed in greater detail (Schonleitner et al., 2017). The choice of technique used depends on the parameter to be measured, and attention should be paid to how the mechanical stress is affecting the cardiomyocyte (load free, stretch, shear etc.) reviewed in Chen-Izu and Izu (2017).

The effect on NO-regulated mechano-transduction/MCT in pathological conditions such as atrial fibrillation in which NO production is dramatically decreased (Reilly et al., 2016) remains to be fully investigated. There is some evidence, however, to suggest that changes to the mechanical environment of the cardiomyocyte are a key element of many disease pathologies. For example, mitral regurgitation causes shear stress in the atria and may thereby provide a mechanism for the development of atrial fibrillation (Grigioni et al., 2002) by affecting ion channel activity. Fibrosis in heart failure and cardiomyopathy can also stiffen the contractile mechanics of the heart (Fatkin and Graham, 2002), whist compromise in NO production is also seen in these disease states (Kelly et al., 1996; Kass et al., 2012). There are indications from recent literature that disease models have limited capacity to respond to increased challenge to the mechanical environment. For example, in atrial cardiomyocytes isolated from rats following the induction of atrial dilatation by trans-aortic constriction, patch clamping showed the ability of voltage gated potassium channels to respond to shear stress was reduced (Boycott et al., 2013). Similarly, subjecting cardiomyocytes from the *mdx* mouse lacking dystrophin to hypo-osmotic solutions to induce membrane stretch caused increased cytosolic Ca^2+^ only in the *mdx* cardiomyocytes (Leijendekker et al., 1996). Finally, experiments using cardiomyocytes plated on to substrates of increasing stiffness showed that obese diabetic rats progressing to heart failure had blunted Ca^2+^ handling with intrinsically stiffer cells due to increased myotubular networks (van Deel et al., 2017).

The extent to which NO can affect these processes and whether NO and stiffness changes work via independent biological signaling mechanisms remains to be investigated. The discovery of a synergistic link between the two would be of great clinical importance when trying to understand and prevent cardiac disease.

## Author Contributions

HB conceived the manuscript, wrote the first draft, drew the first draft figures edited, and approved the final draft. M-NN and BV contributed to sections and figures to the first draft and edited subsequent drafts. PR edited all drafts, wrote discussion and drew figures. KG edited final draft. All authors contributed to the article and approved the submitted version.

## Conflict of Interest

The authors declare that the research was conducted in the absence of any commercial or financial relationships that could be construed as a potential conflict of interest.

## Abbreviations

AKT: protein kinase B
AMPK: AMP-activated protein kinase
ANP: atrial natriuretic peptide
APD: action potential duration
BH_4_: Tetrahydrobiopterin
BNP: brain natriuretic peptide
CaMKII: calcium-calmodulin Ca^2+^/calmodulin-dependent protein kinase II
Ca_*v*_1.2: voltage dependent calcium channel-1.2
CICR: Ca^2+^ induced Ca^2+^ release
DCG: dystrophin-glycoprotein complex
eNOS: endothelial nitric oxide synthase
ERK: extracellular signal-regulated kinase
FAK: focal adhesion kinase
IL-6: Interleukin-6
I_*Na,L*_: late inward sodium current
iNOS: inducible nitric oxide synthase
JNK: c-JunN-terminal kinase
K_*v*_1.5: voltage dependent potassium channel-1.5
LIM domain: lin-11, islet-1, mec-3 domain
L-NAME: ng-nitroarginine methyl ester
LPP: lipoma-preferred partner
LTCC: L-type calcium current
MCT: mechano-chemo-transduction
MEK: mitogen-activated protein kinase kinase
mir31: micro-RNA-31
MLP: muscle LIM protein
Na_*v*_1.5: voltage dependent sodium channel-1.5
NCX: sodium–calcium exchanger
nNOS: neuronal nitric oxide synthase
NO: nitric oxide
NOS: nitric oxide synthase
PI3K: phosphoinositide 3-kinase
PKC-α: protein kinase C- α
PKG: protein kinase G
RyR: ryanodine receptor
SERCA: sarco-endoplasmic reticulum Ca^2+^ ATPase
sGC: guanylate cyclase
SNAP: S-nitroso -N-acetylpenicillamine
SR: sarcoplasmic reticulum
TNFα: tumor necrosis factor- α
TRPC6: transient receptor potential cation channel C6.
